# Editorial Note: Data augmentation-assisted deep learning of hand-drawn partially colored sketches for visual search

**DOI:** 10.1371/journal.pone.0344760

**Published:** 2026-03-12

**Authors:** The PLOS One Editors

## Notice of Republication

The *PLOS One* Editors issue this Editorial Note to inform readers that an undisclosed competing interest between the Academic Editor and the authors was identified after the publication of [1].

PLOS reviewed this matter and concluded that the publication of [1] is supported based on independent expert input that was not affected by the competing interest concerns. PLOS regrets that the issues were not addressed prior to the publication of [1].

This article [1] was republished on February 25, 2026, to replace Figures 1-3, 7, 9, and 10 due to a permissions issue. The original published versions of these figures included content that was published previously by other sources [2–4] which include images that are not available under a CC BY license. The republished article also includes new S1-S2 Files. Please download this article again to view the correct version.
